# Landscape Analysis of Neurodevelopmental Comorbidities in Newborn Screening Conditions: Challenges and Opportunities

**DOI:** 10.3390/ijns10010004

**Published:** 2024-01-04

**Authors:** Zohreh Talebizadeh, Valerie Hu, Monir Shababi, Amy Brower

**Affiliations:** 1 American College of Medical Genetics and Genomics, Bethesda, MD 20814, USA; abrower@acmg.net; 2 Department of Biochemistry and Molecular Biology, The George Washington University School of Medicine, Washington, DC 20037, USA; 3 Independent Research Consultant, Columbia, MO 65203, USA

**Keywords:** newborn screening, neurodevelopmental disorders, common data elements

## Abstract

Newborn screening (NBS) is a large-scale public health program in the US that screens 3.8 million newborns for up to 81 genetic conditions each year. Many of these conditions have comorbidities, including neurodevelopmental disorders (NDDs). These comorbidities can have a significant impact on health outcomes across the lifespan. Most screened conditions are inborn errors of metabolism. PKU, the first condition identified by NBS, is an inherited metabolic disorder that can cause developmental delays and intellectual/developmental disabilities if not treated. The Newborn Screening Translational Research Network (NBSTRN) is a program that has been funded by the National Institute of Child Health and Human Development since 2008. NBSTRN is charged with developing, maintaining, and enhancing tools, resources, and expertise supporting NBS research. One of the tasks led by NBSTRN is to provide direction for developing question/answer sets used in the Longitudinal Pediatric Data Resource (LPDR) to create consensus-based and standardized common data elements (CDEs) for NBS conditions. There is growing interest in the NBS community in assessing neurodevelopmental trajectories through long-term follow-up studies. This could be streamlined by employing uniform CDEs. To address this unmet need, we conducted a landscape analysis to (1) explore the co-occurrence of NDD-related comorbidities and NBS conditions using text mining in MedGen, (2) compile a list of NDD-related CDEs from existing repositories as well as LPDR data dictionaries, and (3) identify challenges and knowledge gaps hindering the early identification of risks for NDDs in NBS conditions. Our findings can inform future efforts toward advancing the research infrastructure for this established public health program. The renewed awareness of the risk of NDDs after a positive NBS and diagnosis could lead to improved treatment guidelines for mental health conditions.

## 1. Introduction

Neurodevelopmental disorders (NDDs) are a group of conditions that affect a child’s learning, behavior, and development. They can include autism spectrum disorder (ASD), attention deficit hyperactivity disorders, intellectual/developmental disabilities (IDDs), communication disorders, behavioral disorders, and motor disorders. NDDs are often diagnosed in early childhood, but some may not be identified until later in life.

Early detection and intervention are essential for improving the outcomes of children with NDDs. Early intervention can help children learn and develop skills, and it can also help families cope with the challenges of raising a child with a disability.

Newborn screening (NBS) is a large-scale public health program in the US that screens 3.8 million newborns for up to 81 genetic conditions each year. Many of these conditions have comorbidities, including NDDs and IDDs. These comorbidities can have a significant impact on health outcomes across the lifespan. Most screened conditions are inborn errors of metabolism. PKU, the first condition identified by NBS, is an inherited metabolic disorder that can cause developmental delays and IDD if not treated.

Over the past decade, the Newborn Screening Translational Research Network (NBSTRN), led by the American College of Medical Genetics and Genomics under a contract from the Eunice Kennedy Shriver National Institute of Child Health and Development, has developed the Longitudinal Pediatric Data Resource (LPDR), a data tool that captures, stores, analyzes, visualizes, and shares genomic and phenotypic data over the lifespan of NBS-identified newborns to facilitate understanding of genetic disease and to assess the impact of early identification and treatment [1,2]. LPDR has the potential to be used for assessment of NDD-related outcomes, as it contains data from newborns who have been screened for NDD-related conditions. However, it is not yet known to what extent LPDR contains related question-and-answer sets.

There is growing interest in the NBS community in assessing neurodevelopmental trajectories through long-term follow-up studies. This could be streamlined by employing consensus-based and standardized common data elements (CDEs). To address this unmet need, we conducted a landscape analysis to identify challenges and opportunities.

We undertook an effort to (1) estimate the proportion of NBS conditions with documented NDD-related comorbidities using text mining in MedGen, (2) gather a list of NDD-related CDEs from existing repositories as well as LPDR data dictionaries, and (3) pinpoint knowledge gaps where additional information is needed to identify NDDs at the earliest possible times after NBS. The findings from this landscape analysis will inform future efforts to advance the research infrastructure for studies on neurodevelopmental trajectories for NBS conditions.

## 2. Methods

### 2.1. Prevalence of NDDs in NBS Conditions

To explore the prevalence of NDDs in NBS conditions, we conducted text mining of clinical information in MedGen [3,4]. MedGen is a database of medical terms and concepts that is maintained by the National Library of Medicine. It is a resource used by clinicians and researchers to find information about specific diseases and conditions. We manually reviewed the information included for each NBS condition and recorded any NDD-related terms.

We employed a systematic manual inspection of NDD-related terms within the clinical descriptions of NBS conditions in MedGen. Two researchers (M.S. and Z.T.) independently reviewed the clinical information for each NBS condition, noting any terms suggestive of NDDs. We focused on terms associated with established NDD diagnoses, but also included clinically relevant terms, which are frequently observed in individuals with certain NDDs.

MedGen information for each NBS condition was reviewed to identify NDD-related terms noted under two MedGen sections: “Definition or Description” and “Additional Description”. The information for these sections is extracted from different resources including GeneReviews, MedlinePlus, and OMIM (Online Mendelian Inheritance in Man). The results were documented in a spreadsheet, which includes NBS conditions and identified NDD-related terms. The keywords were check-marked if they were mentioned in the corresponding source for that specific condition. Similar NDD keywords were merged to reduce repetition. The identified terms were classified into domains and subdomains.

### 2.2. NDD-Related CDEs

To identify existing CDEs that can be used for NDDs, we searched existing CDE repositories, including NIH CDE repositories and Brain Code [5,6]. We classified the NDD-CDEs into basic, screening, and diagnostic categories. Basic CDEs are the most basic and straightforward. They ask simple questions, such as “Does the person have NDD?” and “If yes, what type of NDD?” The answer to one of these questions is typically yes or no, or a single word or phrase. Screening CDEs are used to screen for NDD. They ask more detailed questions about a person’s development and behavior. The answer to these questions can be yes, no, or a number. Screening CDEs can be used to identify people who may be at risk for NDD, but they cannot be used to diagnose NDD. Diagnostic CDEs are used to diagnose NDD. They ask the most detailed questions about a person’s development and behavior. The answers to these questions can lead to a diagnosis of a specific NDD, such as ASD or attention deficit hyperactivity disorder. Diagnostic CDEs are used by healthcare professionals to make a diagnosis of NDD.

### 2.3. NDD-Related CDEs in LPDR Projects

Data dictionaries from the projects stored in the LPDR platform were reviewed to identify NDD-related CDEs. The identified CDEs were classified into three categories: basic, screening, and diagnostic. Screening and diagnostic CDEs were further classified based on the developmental domain into six categories: cognitive, communication, social emotional, motor, ASD, and others.

## 3. Results

We identified 36 NDD-related terms from MedGen, as shown in Table 1. These terms were categorized into six domains: atypical development, cognitive, social-emotional, language and communication, motor, and others. Each domain had multiple subdomains (ranging from two to eight). For instance, the language and communication domain included subdomains related to loss of communication skills and speech issues or difficulties.

Table 2 provides a list of NDD-related terms identified in MedGen for NBS conditions. The prevalence of each domain and subdomain was calculated by dividing the number of NDD-related terms in each domain or subdomain by the total number of conditions. To assess potential differences between core and secondary conditions, similar calculations were performed separately for each category. Overall, the prevalence of each domain in core NBS conditions appeared to be comparable with that in secondary NBS conditions. A more in-depth description of our estimation, including the prevalence for each condition, is provided in Supplemental Table S1.

In reviewing the existing CDE repositories, we identified 198 NDD-related CDEs or questionnaires from NIH repositories that could be applied to neurodevelopmental disorders. A list of these CDEs is provided in Supplemental Table S2. Lists of relevant CDEs identified in LPDR data dictionaries are given in Table 3 and Table 4. A complete list of the question-and-answer sets is provided in Supplemental Table S3.

## 4. Discussion

A number of variables, including treatment effects and knowledge about natural history, are needed to better understand the developmental trajectories of NBS conditions [7]. Long-term follow-up studies can provide such insights [8]. However, the lack of standardized developmental assessments and CDEs makes it challenging to collect uniform information across multiple studies in order to achieve sufficient sample sizes for a meaningful assessment. Our landscape analysis underscores these challenges, but it also highlights the potential for leveraging existing resources, such as basic NDD-related data points in LPDR projects, for secondary data analysis. Additionally, in the absence of a universally accepted assessment protocol for NDDs in NBS conditions, basic information may be collected using screening instruments. While this information is not too specific, it can serve as a baseline, particularly for cases that undergo gene therapy to document pre-procedure developmental status.

Our text mining highlights the need to conduct a cross-sectional comparison of different resources to provide a comprehensive description of NDD profiles for each NBS condition from various perspectives. While MedGen is a valuable resource, we encountered several cases with different information when comparing the results from different sections (i.e., Definition and Additional Description). For example, in the “Definition” section obtained from GeneReviews for a given NBS condition, untreated individuals have been reported to experience a plateauing of cognitive development, followed by the loss of developmental milestones. In contrast, the “Additional Description” section extracted from MedlinePlus lists developmental delay and loss of developmental milestones as symptoms of this condition without specifying their relation to treatment. This suggests that there is a need to consolidate information from different sources to provide a more complete and more accurate picture of the NDD profiles associated with each NBS condition.

Reynolds et al. [7] estimated 65% for the risk of developmental delay in NBS conditions using a matrix applied on 29 conditions automatically eligible for early intervention across states. Our estimate of the prevalence of developmental delays (42%) is lower than this report, which reflects differences in methodologies and inclusion criteria.

The effective implementation and utilization of NBS-derived NDD assessments requires engaging a diverse range of stakeholders, including healthcare providers, researchers, policymakers, parents, and patients. It is important to understand and address the unique needs and preferences of each stakeholder group to ensure that these assessments are relevant, accessible, and useful [9].

Variation in the spectrum and severity of NDD-related symptomology makes it especially challenging in the case of rare conditions to identify the most relevant disease-specific screening and or diagnostic assessment protocols. Alternatively, surveillance protocols such as the one promoted by the American Academy of Pediatrics (AAP) may serve as an intermediate solution to collect baseline outcome data for NBS. Surveillance is the ongoing process of monitoring a child’s development and identifying any potential problems. It involves the use of a standardized test to assess a child’s development in a specific area, such as language, motor skills, or social-emotional development. The AAP recommends that all children be screened for developmental delays at the 9-, 18-, and 30-month well-child visits [10]. If a child is identified as being at risk for an NDD, further evaluation may be needed. This may include a comprehensive assessment by a team of professionals, such as a pediatrician, psychologist, and occupational therapist.

Different stakeholders may rely on different sources of information when making decisions related to NBS. For example, clinicians might refer to databases like MedGen or NewSteps [11], while parents may turn to resources developed by patient advocacy organizations such as NORD [12] and Global Genes [13] or general-purpose health information resources like MedlinePlus [14]. While access to accurate and comprehensive clinical information is crucial for all stakeholders involved, the specific resources preferred often differ due to varying needs and expertise. For example, PubMed [15] is widely used for peer-reviewed research articles, ClinGen [16] for curated genetic variants, and specialized databases like OMIM [17] and GeneReviews [18] for in-depth summaries of specific disorders, while resources like GARD (Genetic and Rare Diseases Information Center) [19] may be used for disease summaries and treatment options, point-of-care decision support tools, and drug databases. To provide a comprehensive description of NDD profiles for each NBS condition from various perspectives, it is necessary to conduct a cross-sectional comparison of different resources.

Patient-reported outcome (PRO) data [20] can play a valuable role in assessing the impact of NDDs on individuals and their families. PRO data can be used to inform the development of NBS-derived NDD assessments, track the progression of NDDs over time, and evaluate the effectiveness of interventions.

By engaging stakeholders and using PRO data, we can develop and implement NBS-derived NDD assessments that are relevant, useful, and informative. This will help to improve the quality of life of children and adults with NDDs and their families.

The present landscape analysis offers a holistic overview of neurodevelopmental disorder profiles within the context of NBS conditions, encompassing various developmental domains, prevalence rates, and potential actionable CDEs for future research and clinical applications. Findings from this landscape analysis can inform future efforts toward advancing the research infrastructure for studies of neurodevelopmental trajectories for NBS conditions.

## 5. Limitations

While text mining in MedGen provided a valuable initial estimation of the proportion of NBS conditions with documented mentions of NDD-related comorbidities, this approach cannot definitively measure prevalence. Future epidemiological studies are needed to expand our understanding of the full NDD profiles within NBS conditions and validate the initial insights provided by text mining. Additionally, reliance on US-centric resources limited the generalizability of our findings to other healthcare systems, and we potentially missed relevant information specific to other regions.

## 6. Future Directions

The present landscape analysis sets the stage for future exploration in the following directions.

- Improving text mining accuracy and generalizability: Future research might prioritize investigating approaches to address data biases and integrate non-US resources in text mining for identifying NDD-related comorbidities in NBS conditions, thereby capturing a more global perspective.
- Deepening multi-domain integration: Future studies could explore strategies for integrating the framework with other relevant domains such as neuro-muscular and neuro-degenerative. This could involve harmonizing data formats, developing cross-domain data-exchange protocols, and incorporating multi-domain CDEs. Such expanded data capture and analysis could potentially improve diagnosis, early intervention, and patient care for comorbidities in NBS conditions.
- Strengthening global applicability: Future research directions may involve aligning NDD-CDEs with established international standards like the WHO ICF [21] and regional CDE sets used in specific healthcare systems. Additionally, assessing data dictionaries adopted by existing international initiatives focused on rare disease (RD) registries and NBS follow-up, such as the EU-RD platform [22,23], could inform CDE harmonization. Expanding the scoping to non-US resources would foster a more comprehensive understanding of NDD co-occurrence in NBS conditions and maximize interoperability of NBS/RD registry data across borders.

## Figures and Tables

**Table 1:** Summary of NDD-related terms associated with NBS conditions. This table offers a summary of NDD-related terms associated with NBS conditions identified in MedGen through text mining. The terms are categorized into six developmental domains, with similar terms merged to minimize redundancy. For instance, developmental regression and loss of developmental milestones are grouped under subdomain 1b.

Domains	NDD-Related Terms		
**1. Atypical Development**			
**1a.**	Developmental delay	Delayed development	Progressive problems with development
**1b.**	Developmental regression	Loss of developmental milestones	
**2. Cognitive**			
**2a.**	Cognitive impairment	Plateauing of cognitive development	Cognitive developmental delay
**2b.**	Intellectual disabilities	Deficit/impairment/poor intellectual function	
**2c.**	Learning disability	Learning difficulty	
**2d.**	Mental retardation	Mental health disorder	
**2e.**	Loss of memory		
**2f.**	Dementia		
**2g.**	Progressive cognitive deterioration		
**2h.**	Decline in intellectual function		
**3. Social-emotional**			
**3a.**	Attention-deficit/hyperactivity disorder		
**3b.**	Behavioral problems/abnormalities/issues		
**3c.**	Irritability		
**3d.**	Aggressiveness		
**3e.**	Neuropsychiatric symptoms (hyperactivity, delusions, disorientation, restlessness, hallucinations)		
**3f.**	Psychosis/psychiatric problems/disorder		
**4. Language and Communication**			
**4a.**	Loss of communication skills		
**4b.**	Speech issues/difficulties/defects		
**5. Motor**			
**5a.**	Delayed psychomotor development		
**6. Others**			
**6a.**	Failure to thrive	Growth impairment/failure	Restricted/poor/slow/slower-than-normal growth
**6b.**	Neurological deficit/symptoms/impairment	Neurologic crisis	Neurological abnormalities
**6c.**	Seizures	Epilepsy	
**6d.**	Loss of consciousness		

**Table 2:** Comparison of NDD-related terms in core and secondary NBS conditions. This table presents the prevalence of NDD-related terms identified through text mining within each domain for core and secondary NBS conditions. Refer to Table S1 for detailed calculation methods.

Domains	All (*n* = 60)	Core (*n* = 36)	Secondary (*n* = 24)
**1. Atypical Development**			
**1a.**	24 (40%)	14 (39%)	10 (42%)
**1b.**	5 (8%)	3 (8%)	2 (8%)
**Total**	**25 (42%)**	**15 (42%)**	**10 (42%)**
**2. Cognitive**			
**2a.**	4 (7%)	3 (8%)	1 (4%)
**2b.**	20 (33%)	10 (28%)	10 (42%)
**2c.**	6 (10%)	4 (11%)	2 (8%)
**2d.**	8 (13%)	2 (6%)	6 (25%)
**2e.**	2 (3%)	0	2 (8%)
**2f.**	2 (3%)	0	2 (8%)
**2g.**	2 (3%)	1 (3%)	1 (4%)
**2h.**	5 (8%)	3 (8%)	1 (4%)
**Total**	**28 (47%)**	**14 (39%)**	**14 (58%)**
**3. Social-emotional**			
**3a.**	1 (2%)	1 (3%)	0
**3b.**	4 (7%)	3 (8%)	1 (4%)
**3c.**	5 (8%)	2 (6%)	3 (13%)
**3d.**	2 (3%)	1 (3%)	1 (4%)
**3e.**	2 (3%)	0	2 (8%)
**3f.**	4 (7%)	3 (8%)	1 (4%)
**Total**	**11 (18%)**	**6 (17%)**	**5 (21%)**
**4. Language and Communication**			
**4a.**	1 (2%)	0	1 (4%)
**4b.**	4 (7%)	1 (3%)	3 (13%)
**Total**	**5 (8%)**	**1 (3%)**	**4 (17%)**
**5. Motor**			
**5a.**	12 (20%)	5 (14%)	7 (29%)
**Total**	**12 (20%)**	**5 (14%)**	**7 (29%)**
**6. Others**			
**6a.**	21 (35%)	15 (42%)	6 (25%)
**6b.**	13 (22%)	4 (11%)	9 (38%)
**6c.**	29 (48%)	15 (42%)	14 (58%)
**6d.**	1 (2%)	1 (3%)	0
**Total**	**40 (67%)**	**23 (64%)**	**17 (71%)**

**Table 3:** NDD-related instruments in LPDR. The screening/diagnostic NDD-related CDEs are listed based on the developmental domain assessed by each instrument.

Developmental Domain	Instrument
Cognitive	Carey Temperament Scales, WJ-EDAC, Early Childhood Toolbox, NIH Toolbox Cognitive Battery, WPPSI/WISC, CTOPP-2
Communication	MacArthur Bates Communicative Development Inventories
Social-emotional	ASIEP, Brief-P, CELF-P2, SRS-2
Motor	AIMS
Autism spectrum disorder	ADOS, M-CHAT
Other	PEDS, Child Behavior Checklist, Eye Tracking Protocol, Infant Toddler Checklist

**Table 4:** Types of NDD-related CDEs in LPDR. The specific developmental domains that are covered by each project are listed. More details about each LPDR project can be found on the NBSTRN website (www.nbstrn.org).

Project Name	CDE Types	Developmental Domains
eXtraordinarY Babies	Basic, Screening, Diagnostic	Cognitive, Motor, Social-emotional, Communication, ASD, Others
IBEMC	Basic	
SPOT SMA	Basic, Screening	Cognitive, Motor, Social-emotional, Communication
Cure-SMA	Basic, Screening	Motor
PCH	Basic	
WorldwideKrabbeDiseaseRegistry	Basic	
LSD CIG Subgroup	Basic	

## Supplementary Materials

The following supporting information can be downloaded at: https://www.mdpi.com/article/10.3390/ijns10010004/s1. Table S1. NDD-related terms associated with NBS conditions. This table provides a detailed list of NDD-related terms identified, through MedGen text mining, for each NBS condition. Table S2. NDD-CDEs identified in NIH CDE repositories. This table provides a comprehensive list of NDD-related CDEs, including their names, question texts, value types, variable descriptions, and permissible values. Table S3. NDD-CDEs in LPDR data dictionaries. This table provides a comprehensive list of NDD-related CDEs identified in the LPDR data dictionaries, including their names, question texts, value types, variable descriptions, and permissible values. More details about each LPDR project can be found on the NBSTRN website (www.nbstrn.org).
